# Betulin Stimulates Osteogenic Differentiation of Human Osteoblasts-Loaded Alginate–Gelatin Microbeads

**DOI:** 10.3390/bioengineering11060553

**Published:** 2024-05-30

**Authors:** Mehmet Ali Karaca, Derya Dilek Kancagi, Ugur Ozbek, Ercument Ovali, Ozgul Gok

**Affiliations:** 1 Department of Medical Biotechnology, Institute of Health Sciences, Acibadem Mehmet Ali Aydinlar University, 34752 Istanbul, Turkey; mehmetalikaraca0@gmail.com; 2 Acibadem Labcell Cellular Therapy Laboratory, 34752 Istanbul, Turkey; derya.kancagi@acibademlabcell.com.tr (D.D.K.); ercument.ovali@acibadem.com (E.O.); 3 Medical Genetics Department, School of Medicine, Acibadem Mehmet Ali Aydinlar University, 34752 Istanbul, Turkey; ugur.ozbek@acibadem.edu.tr; 4 Department of Biomedical Engineering, Faculty of Engineering and Natural Sciences, Acibadem Mehmet Ali Aydinlar University, 34752 Istanbul, Turkey

**Keywords:** betulin, alginate–gelatin microbeads, osteogenic differentiation, human fetal osteoblast cells

## Abstract

Osteoporosis, a terminal illness, has emerged as a global public health problem in recent years. The long-term use of bone anabolic drugs to treat osteoporosis causes multi-morbidity in elderly patients. Alternative therapies, such as allogenic and autogenic tissue grafts, face important issues, such as a limited source of allogenic grafts and tissue rejection in autogenic grafts. However, stem cell therapy has been shown to increase bone regeneration and decrease osteoporotic bone formation. Stem cell therapy combined with betulin (BET) supplementation might be adequate for bone remodeling and new bone tissue generation. In this study, the effect of BET on the viability and osteogenic differentiation of hFOB 1.19 cells was investigated. The cells were encapsulated in alginate–gelatin (AlGel) microbeads. In vitro tests were conducted during the 12 d of incubation. While BET showed cytotoxic activity (>1 µM) toward non-encapsulated hFOB 1.19 cells, encapsulated cells retained their functionality for up to 12 days, even at 5 µM BET. Moreover, the expression of osteogenic markers indicates an enhanced osteo-inductive effect of betulin on encapsulated hFOB 1.19, compared to the non-encapsulated cell culture. The 3D micro-environment of the AlGel microcapsules successfully protects the hFOB 1.19 cells against BET cytotoxicity, allowing BET to improve the mineralization and differentiation of osteoblast cells.

## 1. Introduction

Natural bone tissue is composed of organic and inorganic materials and mixed cellular structures (osteoprogenitors, osteoblasts, osteocytes, and osteoclasts) [1]. New bone formation, which is known as the ossification process, is initiated with the degradation of old bone and removal of dead cells by osteoclasts, followed by the condensation of mesenchymal stem cells, the formation of osteoprogenitor cells, and their differentiation into osteoblasts, which can secrete proteins and calcify the extracellular matrix (ECM) environment [2]. Imbalance during the ossification process results in bone diseases, such as osteopenia and osteoporosis. Osteoporosis, which is a type of bone fracture, occurs due to the alteration of bone composition and structure by aging processes. Today, anti-resorptive therapy and bone anabolic drugs have been used to decrease osteoporosis inside the bone structure [3]. However, the long-term usage of these drugs leads to side effects such as multi-morbidity in elderly people or patients. Other therapies for osteoporosis treatment include allogenic and autogenic tissue grafts, and stem cell therapy. Limited sources of allogenic grafts and tissue rejection of autogenic grafts restricts transplant therapy [4]. Bone tissue engineering-based products, such as stem cell-loaded materials, are promising tools for bone grafting in osteoporosis.

Bone tissue engineering strategies have been used to mimic bone tissue and provide an efficient cellular therapy for the treatment of bone defects and diseases [5]. However, the absence of a vascular structure and the low sustainability of cell viability inside the scaffold decrease therapeutic efficiency after transplant. Polymeric materials provide better environmental conditions for cell viability, proliferation, and differentiation, compared to other biomaterials. A bone-like polymeric scaffold for the encapsulation of cells initiates their differentiation from mesenchymal stem cells to osteoblasts [6]. The development of cell-loaded microbeads with various polymeric materials might allow for a sufficient mass transfer, keeping cells safe after transplant, so that building blocks can be made from microbeads.

Alginate, a commonly used natural biomaterial, is the major component of polymeric scaffolds in microbeads suitable for tissue engineering and cell therapy applications for the heart [7], bone [8], skeletal tissue [9], testis [10], liver [11], and chondrocytes [12]. The fabrication of alginate-encapsulated islet cells is an attractive way to enhance therapeutic efficiency for and the life quality of type 1 diabetes patients without the administration of immuno-suppressants [13]. Different alginate polymers, e.g., peptide complexes and blended forms, have been used to modify the encapsulation efficiency and to increase the cellular adhesion and proliferation of osteoblasts [14]. Additionally, the morphology of the encapsulated cells and their behavior, and the release kinetics of alginate microbeads, may be affected by the modification of cations or by variations in instrumental parameters [15]. Also, environmental conditions of cell-loaded alginate microbeads change the microbeads’ stability and alginate degradation [16,17].

Strategies that involve the use of hybrid polymers for microbead fabrication have attracted a lot of attention in recent years due to their beneficial features. Alginate–gelatin (AlGel) blend formulations enhance cell viability and differentiation inside the microsphere [6]. Since gelatin, the denatured form of collagen type-1 protein inside the ECM, contains the arginine–glycine–asparagine (RGD) peptide sequence in its structure, its incorporation into scaffolds improves cell adhesion, differentiation, and proliferation [18]. Although the poor mechanical properties of gelatin limit the construction of three-dimensional (3D) scaffold structures for culturing cells, its presence increases cell adhesion in different types of polymeric scaffolds [19,20]. A blend formulation of alginate and gelatin enhances the mechanical properties of cell-loaded alginate microspheres [20]. Another study showed that gelatin in the alginate mixture leads to improved cell adhesion properties because of the RGD (Arg–Gly–Asp) residue inside the microbeads [19]. An alginate–gelatin mixture was tested with various cell types, e.g., rat cardio myoblasts [21,22,23,24], H9C2 [22], U937 [25,26], HepG2 [27], Hatac [20], and human endothelial cells (HUVEC) [24].

Cell-loaded alginate–gelatin microspheres are promising for the enhancement of the mechanical properties of microbeads, the maturation of the osteoblast cell, and the regeneration of bone fractures. Firouzi et al. conducted a study of AlGel crosslinked microcapsules (ADA-GEL) with a tunable stiffness and improved stability compared to alginate hydrogel [28]. The effect of the mechanical properties on osteogenic differentiation inside the AlGel microcapsule might be associated with mitogen-induced protein kinase activation downstream of the RhoA-Rho-associated protein kinase (ROCK) signaling pathway [29,30]. Additionally, stiffer AlGel microcapsules increase the mineral deposition and osteogenesis potential. Moreover, metabolites such as ATP are important for the cellular differentiation of mesenchymal stem cells [31]. Respiration of the cell and oxidative stress inside the microbeads might be regulated via a blend formulation of the alginate microbeads. Gelatin inside the alginate microbeads seems to enhance the aerobic respiration of the human adipose-derived mesenchymal stem cells [32].

The in vitro differentiation of mesenchymal stem cells into osteocytes, adipocytes, and chondrocytes is regulated by environmental factors such as the interaction between biomaterials and stem cells and supplements inside the osteogenic medium (fetal bovine serum, ascorbic acid, beta-glycerophosphate, dexamethasone, etc.). The interaction of biomaterials with stem cells activates various signaling pathways, such as TGF-β/BMPs, the Notch receptor, Wnt/β-catenin, Hedgehog, and mitogen-activated protein kinase (MAPK) [33]. This activation promotes the cell adhesion, proliferation, and osteogenic differentiation of mesenchymal stem cells. The adhesion of mesenchymal stem cells onto biomaterial also activates intracellular pathways, such as the MAPK signaling pathway [34]. In this pathway, maturation of the stem cells is initiated by the adhesion of cells, followed by proliferation, and completed by differentiation [35]. The maturation process is followed by the activation of extracellular signal-regulated kinases (ERK) pathways (7 to 11 days), and the activation of Jun N-terminal kinases (JNK) pathways and p38, which regulate the late stages of cell differentiation and apoptosis. The maturation process ends with calcium depositions and ECM synthesis (collagen) (13 to 17 days).

Betulin (BET) is a type of triterpenoid extracted from betula papyrifera that has been used for the treatment of microfractures and dislocated bones in traditional medicine [36]. BET has various pharmacological properties: anti-tumor, anti-inflammatory, antiviral, antibacterial, and osteogenic activities [37,38]. The anti-tumor effect of betulinic acid has been demonstrated in various cell lines, e.g., melanoma, neuroblastoma, medulloblastoma, glioblastoma, head and neck cancer, ovarian carcinoma, cervix carcinoma, lung carcinoma, and leukemia [39]. The effect of BET on cancer cells might be correlated with the induction of apoptosis via loss of mitochondrial membrane potential. The osteogenic potential of BET was shown by various signaling cascades, especially the activity of c-JNK and ERK [40]. A recent study showed that BET activates the JNK, ERK1/2, and mTOR kinase-dependent signaling pathways and enhances osteogenesis of the human fetal osteoblast cell line (hFOB 1.19) [41].

In this work, we investigated the effect of BET on the differentiation and mineralization of hFOB 1.19 cells that were encapsulated in polymeric microbeads composed of crosslinked alginate polymer chains with gelatin (AlGel). For that purpose, hFOB 1.19 cell-loaded AlGel microbeads were prepared with an electrostatically assisted spraying system. The osteo-inductive effect of BET on hFOB 1.19 cells in this 3D microenvironment was evaluated in a dose-dependent manner via alizarin red S staining method and osteopontin production (OPN) measurements during 12 days of incubation.

## 2. Materials and Methods

### 2.1. Materials

A solution containing 4% *w*/*w* alginate (50% mannuronate units, Sigma-Aldrich, low viscosity, A1112, Taufkirchen, Germany) and 0.4% *w*/*w* gelatin (from bovine skin, type B, Sigma-Aldrich, Taufkirchen, Germany) in dH_2_O was prepared by stirring it overnight at room temperature (RT). Alginate–gelatin mixtures (AlGel) were thus obtained. Calcium chloride (CaCl_2_, Merck, Taufkirchen, Germany) at a concentration of 75 mM was used as a crosslinking solution. Nozzles (0.35 mm) were incubated in a sodium citrate solution (85 mM) to prevent the clogging of the system when running the experiment. The encapsulation systems were cleaned with ethanol (70%). Then, the encapsulation systems and the AlGel and CaCl_2_ solutions were sterilized via UV treatment for 2 h. A betulin (BET) stock solution was prepared in DMSO (Sigma-Aldrich, Taufkirchen, Germany), and 1 mL aliquots were stored at 4 °C. The concentration of DMSO in all the cell culture experiments was below 0.01%. The triterpenoid stock solution was prepared prior to use in the cell culture experiment.

### 2.2. Cell Culture Experiments

The human osteoblast cell line hFOB 1.19 (ATCC, CRL-11372, Manassas, VA, USA) was cultured in Dulbecco’s Modified Eagle’s Medium (DMEM, Phenol Red Free, Gibco, New York, NY, USA), supplemented with 10% fetal bovine serum (FBS, Gibco, New York, NY, USA) and1% penicillin-streptomycin (PSA, Gibco, New York, NY, USA) at a temperature of 37 °C under a humidified 5% CO_2_ atmosphere.

The effect of BET on osteoblast cell differentiation was investigated in an osteogenic medium supplemented with 1% FBS and 1% PSA. The osteogenic medium was prepared with the addition of 10 mM of beta-glycerophosphate (β-GlyP, Sigma-Aldrich, Taufkirchen, Germany) and 50 µg/mL of ascorbic phosphate–magnesium salt (AA, Sigma-Aldrich, Taufkirchen, Germany). Media were exchanged every two days; the hFOB 1.19 cells were cultured over 12 days.

### 2.3. Fabrication of Cell-Loaded Microbeads

For the sterilization of the encapsulator’s operation systems (Nisco Encapsulator VAR V1 LIN-0043, Nisco Engineering AG, Zurich, Switzerland), ethanol (70%) was run before the cell culture experiment. hFOB 1.19 cells were trypsinized from the culture flask, and cell numbers were calculated with a cell-counting device (BioRad, Hercules, CA, USA). hFOB 1.19 cells (4 × 10^6^ cells/mL) were dissolved with a UV-sterilized AlGel polymer solution and put into a syringe (Becton Dickinson, BD, Bergen County, NJ, USA). Then, the cell–polymer mixture was run in the system until it was fully loaded (cables and 0.35 mm tip). The electrode was dipped into the crosslinking solution, and the voltage was adjusted to 6 kVa, which is an optimum value for microbead production. The hFOB 1.19 cell-loaded AlGel microbeads were prepared by electrostatic droplet generation [42,43].

After preparation, the hFOB 1.19 cell-loaded AlGel microbeads (1000 microbeads/mL) were cultivated in DMEM containing 10% FBS and visualized with a fluorescence microscope, after adding fresh medium on days 2, 4, 6, 8, 10, and 12. A total of three microbeads were selected for each group. The diameter of the microbeads was measured with the Zeiss software (Carl Zeiss Microscope, Oberkochen, Germany). The diameter on day zero was considered as 100% (reference value).

### 2.4. Cell Proliferation and Metabolic Activity

The effect of BET on hFOB 1.19 cell proliferation was investigated using the MTS assay kit (Abcam, ab197010, Cambridge, UK). hFOB 1.19 cells (5 × 10^3^ cells/well) were seeded on 96-well plates. After 24 h, the cells were treated with BET at different concentrations (0, 0.01, 0.1, 0.5, 1, and 5 µM) in DMEM containing 1% FBS without the addition of osteogenic supplements [41]. After 96 h, hFOB 1.19 cell proliferation was examined using the MTS kit, according to the manufacturer’s instructions.

The same assay was used to assess the effect of BET on hFOB 1.19 cell proliferation inside the microbeads. The hFOB 1.19 cell-loaded AlGel microbeads were treated with BET (0, 0.01, 0.1, 0.5, 1, and 5 µM) in an osteogenic medium (Section 2.2). On day 12, the hFOB 1.19 cell-loaded microbeads were collected, and the viability of the cells was evaluated (triplicate wells) with the MTS kit, according to the manufacturer’s instructions. The viability in the control well was considered as 100% (reference value).

### 2.5. Live and Dead Assay

The viability of the hFOB 1.19 cell-loaded AlGel microbeads was assessed for cultures with various concentrations of BET (0, 0.01, 0.1, 0.5, 1, and 5 µM) in DMEM with1% FBS, 1% PSA, 10 mM of β-GlyP, and 50 µg/mL of AA, using the Live and Dead cell assay (Abcam, ab115347, Cambridge, UK).

hFOB 1.19 cell-loaded microbeads cultured in 0 µM BET were collected on days 2, 4, 6, 8, 10, and 12, while hFOB 1.19 cell-loaded microbeads with 0.01, 0.1, 0.5, 1, and 5 µM BET were collected on days 6 and 12. Each group of microbeads was suspended (1:1) with 10× Live and Dead cells’ stains. The samples were incubated with the dyes for 10 min at RT. Stained hFOB 1.19 cell-loaded microbeads were visualized with a fluorescence microscope (EVOS^TM^ M5000 Cell Imaging System, ThermoFisher, Bothell, Washington, DC, USA). Viable cells’ numbers, red and green cells, were counted in three microbeads from each sample. The viable cells’ number on day 0 was considered 100% (reference value). To evaluate the effect of the BET treatment, the viable cells’ number reference (100%) was that of 0 µM BET.

### 2.6. Concanavalin A–Alexafluor (ConA-AF488) and DAPI Staining

hFOB 1.19 cell loaded–microbeads which were cultured with various concentrations of BET (0, 0.01, 0.1, 0.5, 1, and 5 µM) in DMEM containing 1% FBS, 1% PSA, 10 mM of β-GlyP, and 50 µg/mL of AA were collected on days 0 and 12 and washed with CaCl_2_ solution three times. Each group of microbeads was suspended with a 20 μg/mL ConA-AF488 solution and incubated for 1 h. After incubation, the microbeads were washed with a PBS solution three times and then stained witha10 µg/mL DAPI solution for 6 min. After washing, the hFOB 1.19 cell-loaded microbeads were visualized with the fluorescence microscope. A total of three microbeads were selected to measure the signal intensity of the ConA-AF488 solution and the DAPI for each group. Intensity values on day 0 were considered as 100% (reference value). To evaluate the effect of the BET treatment, the intensity values’ reference (100%) was that of 0 µM BET.

### 2.7. Osteopontin Production Assay

The ELISA method was used to measure the OPN concentration in the cell culture media of hFOB 1.19 cells and hFOB 1.19 cell-loaded microbeads. hFOB 1.19 cells were seeded in 96-well plates at a density of 3 × 10^4^ cells/well. After 24 h, the cells were treated with different concentrations of BET (0, 0.01, 0.1, 0.5, 1, and 5 µM) in DMEM containing 1% FBS, 1% PSA, 10 mM of β-GlyP, and 50 µg/mL of AA for 12 d; culture media samples were collected on day 12. Similarly, the hFOB 1.19 cell-loaded microbeads were incubated with BET (0, 0.01, 0.1, 0.5, 1, and 5 µM) in DMEM containing 1% FBS, 1% PSA, 10 mM of β-GlyP, and 50 µg/mL of AA for 12 d, and media samples were collected on day 12.

The collected media samples were centrifuged and stored at −80 °C before use in the OPN concentration measurements (OPN human ELISA kit, Abcam, Boston, MA, USA). The OPN concentration in the control well was considered as 100% (reference value).

### 2.8. Dimethylmethylene Blue Assay (DMMB)

A Dimethylmethylene Blue Assay (DMMB) was performed to measure the amount of glycosaminoglycans (GAGs) polysaccharides in the cell culture media of hFOB 1.19 cell loaded–microbeads. The microbeads were incubated with BET (0, 0.01, 0.1, 0.5, 1, and 5 µM) in DMEM containing 1% FBS, 1% PSA, 10 mM of β-GlyP, and 50 µg/mL of AA for 12 d. Culture media samples were collected on day 12. The collected samples were centrifuged and stored at −80 °C before use in the DMMB assay.

Samples were suspended in the DMMB solution. The GAG concentration in the solution is determined from the chondroitin sulfate standard calibration curve. The GAG concentration in the control group was considered as 100% (reference value).

### 2.9. Alizarin Red S Staining

The degree of mineralization of the extracellular matrix of the hFOB 1.19 cells and hFOB 1.19 cell-loaded microbeads was evaluated using 2% alizarin red S (Merck, Darmstadt, Germany) staining. Before staining, the hFOB 1.19 cells and hFOB 1.19 cell-loaded microbeads were incubated with BET (0, 0.01, 0.1, 0.5, 1, and 5 µM) in an osteogenic medium for 12 d.

After treatment with BET, the hFOB 1.19 cells were washed twice with PBS and fixed with a 4% formalin solution. The fixed hFOB 1.19 cells were then washed twice with dH_2_O, stained with alizarin redS for 1 h, and washed again twice with dH_2_O. The stained cells were visualized with the fluorescence microscope.

Similarly, hFOB 1.19 cell-loaded microbeads were collected after treatment with BET and washed with HEPES solution thrice. The microbeads were fixed with a 4% (*v*/*v*) formaldehyde solution at RT. The microbeads were then stained with 2% alizarin redS and incubated for 15 min. The stained microbeads were washed twice with dH_2_O and visualized with the fluorescence microscope.

The alizarin red dye was extracted from the stained microbeads with 10% cetylpyridinium chloride (CPC, Sigma-Aldrich, C0732, Taufkirchen, Germany) in 10 mM sodium phosphate (pH = 7) for 1 h by rotating the plate at RT. After the incubation period, 200 µL samples were transferred into 96-well plates.

The extracted samples were serially diluted for quantification of the matrix calcium deposition. The absorbance of the samples at 562 nm was measured with a microplate reader (FLUOstar Omega, BMG Labtech, Ortenberg, Baden-württemberg, Germany). All measurements for the degree of mineralization were performed in triplicate wells. The mineralization degree in the control well was considered as 100%.

### 2.10. Alkaline Phosphatase Activity

hFOB 1.19 cell-loaded microbeads were incubated with BET (0, 0.01, 0.1, 0.5, 1, and 5 µM) in osteogenic medium for 12 d. After treatment with BET, the hFOB 1.19 cell-loaded microbeads were collected and washed twice with PBS solution. The microbeads were then incubated with BCIP/NBT plus suppress solution (34070, Thermo Fisher, Rockford, IL, USA) for 60 min. Alkaline phosphatase (ALP)-positive microbeads were washed with 4% (*v*/*v*) formaldehyde, then washed twice with buffer. The samples were visualized using the fluorescence microscope.

### 2.11. Statistical Analyses

Statistical analyses were performed using Microsoft Excel software 16.84 (Redmond, Washington, DC, USA). The results were analyzed by running Student’s *t*-test from the averaged data obtained from three independent experiments with a *p*-value < 0.05. The levels of significance were shown at *: *p* < 0.05, **: *p* < 0.01, and ***: *p* < 0.001.

## 3. Results

### 3.1. Morphological Evaluation of hFOB 1.19 Cell-Loaded Microbeads

**Figure 1:**
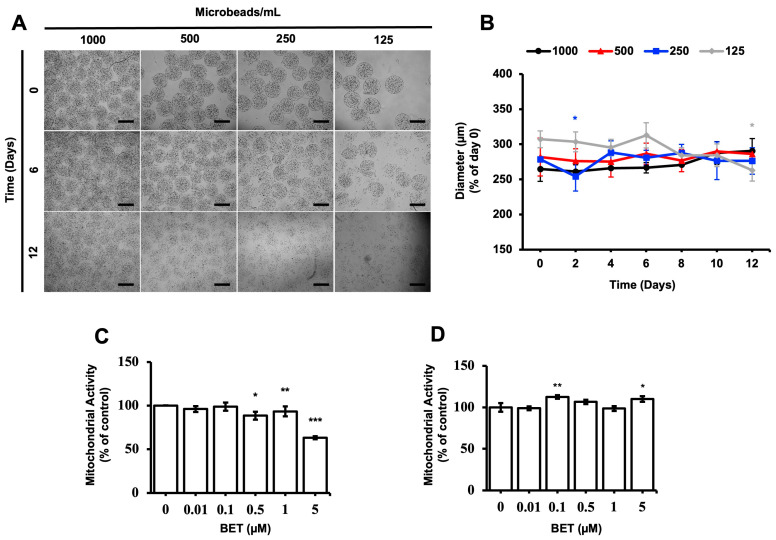
Stability of cell-loaded AlGel microbeads. (**A**) Microscopic visualization of hFOB 1.19 cells inside AlGel microbeads and hFOB 1.19 cells released from microbeads (scale bar: 200 µm). (**B**) Diameter of hFOB 1.19cell-loaded microbeads in DMEM medium during 12 d of incubation. Effect of BET on mitochondrial activity of (**C**) hFOB 1.19 cells cultured in 2D cell culture system on day 6 and (**D**) hFOB 1.19 cells encapsulated in AlGel microbeads on day 12 (1% FBS growth medium supplement with various concentrations of BET) *: *p* < 0.05, **: *p* < 0.01, and ***: *p* < 0.001.

The stability and proliferation of the hFOB 1.19 cells inside the AlGel microbeads were tested with different amounts of microbeads (1000, 500, 250, and 125 microbeads/mL) for 12 d. The microbeads were not stable at 500, 250, and 125 microbeads/mL for the 12 d period (Figure 1A). The diameter of the microbeads was not significantly altered as the days passed, except for the 250 microbeads/mL sample on day 2 and the 125 microbeads/mL sample on day 12 (Figure 1B). As seen from Figure 1A,B, there seems to be no direct correlation between the degradation of the microbeads and changes in diameter, while the degradation behavior is enhanced by decreasing the microbead concentration.

### 3.2. Effect of BET on the Metabolic Activity of hFOB 1.19 Cells

The effect of BET on the metabolic activity of hFOB 1.19 cells was assessed with varying concentrations of BET, using the MTS assay. The mitochondrial respiration of the cells, cell viability, growth rate, and cellular energy capacity (indirect) were evaluated by the MTS assay [44]. Even though cytotoxic activity of BET on the hFOB 1.19 cells was observed at 5 µM BET on day 6, the metabolic activity of the cells was not suppressed when they were inside the microbeads at this concentration of BET on day 12. In addition, a statistically significant decrease of mitochondrial activity was observed at 0.5, 1, and 5 µM BET on day 6, whereas no clear decrease was detected once the cells were inside the AlGel microbeads on day 12 (Figure 1C,D). Thus, it can be stated that the AlGel microbeads are a barrier that reduces the susceptibility of hFOB 1.19 cells to BET.

### 3.3. Effect of BET on Cell Viability of hFOB 1.19 Cell-Loaded AlGel Microbeads

**Figure 2:**
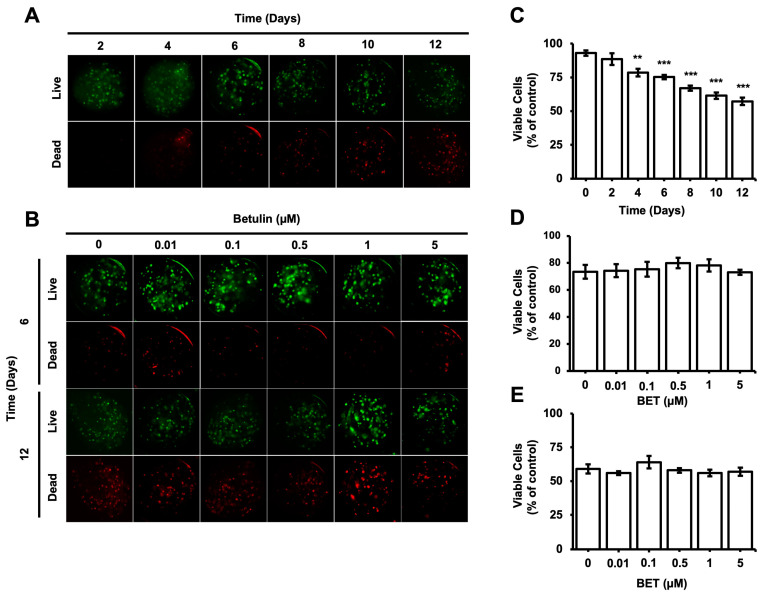
Viability of hFOB 1.19 cells in AlGel microbeads. (**A**) Fluorescence images of live/dead staining for hFOB 1.19 cells in microbeads on days 2, 4, 6, 8, 10, and 12 (scale bar: 100 µm). (**B**) Fluorescence images of live/dead staining for hFOB 1.19 cells encapsulated in AlGel microbeads incubated with various concentration of BET (0 to 5 µM) on days 6 and 12 (scale bar: 100 µm). (**C**) Percentage of viable cells in microbeads on days 2, 4, 6, 8, 10, and 12. Percentages of viable cells encapsulated in AlGel microbeads incubated with different concentrations of BET (0 to 5 µM) on days 6 (**D**) and 12 (**E**). **: *p* < 0.01, and ***: *p* < 0.001.

The viability of cells encapsulated inside the microbeads was evaluated during the incubation period using the Live and Dead cells assay (ab115347, Abcam). According to the fluorescent microscopy images obtained upon proper staining, the percentage of viable cells gradually decreased from 93% on day 0 to 57% on day 12; a statistically significant decrease was observed on day 4 (Figure 2C). The number of dead cells inside the bigger aggregates seems to increase gradually with time. However, the cytotoxic effect of BET on the encapsulated cells remained almost consistent among samples on days 6 and 12 (Figure 2B,D,E).

### 3.4. Alteration of High Mannose-Type Glycans (HM) Levels in Microbeads

**Figure 3:**
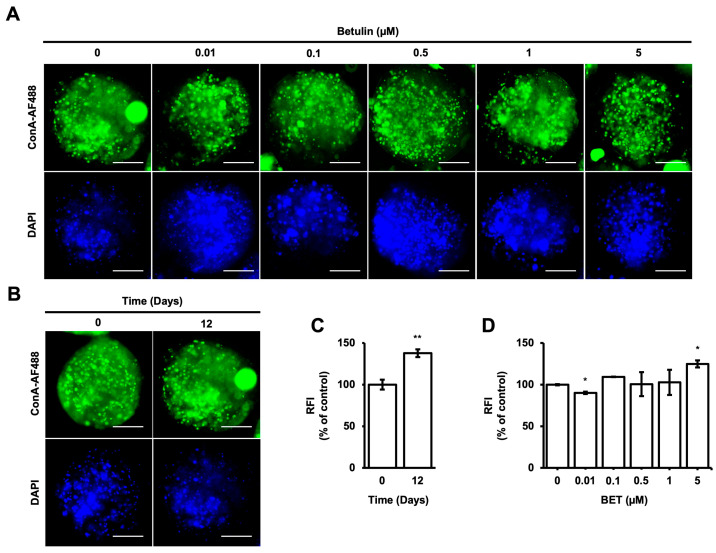
Localization of HMsin hFOB 1.19 cells encapsulated in AlGel. (**A**) Fluorescence images of ConA-AF488- and DAPI-labeled hFOB 1.19 cells encapsulated in AlGel incubated with different concentrations of BET (0 to 5 µM) on day 12 (scale bar: 100 µm). (**B**) hFOB 1.19 cells encapsulated in AlGel on days 0 and 12 (scale bar: 100 µm). (**C**) Relative ConA-AF488 intensities for hFOB 1.19 cells encapsulated in AlGel on days 0 and 12. (**D**) Relative ConA-AF488 intensities for hFOB 1.19 cells encapsulated in AlGel incubated with different concentrations of BET (0 to 5 µM) on day 12. RFI: relative fluorescence intensity. *: *p* < 0.05, and **: *p* < 0.01.

Concanavalin-A, which is a type of lectin, has a strong binding affinity to mannose compared to other sugars. ConA-AF488 has been utilized as a monitoring tool for the evaluation of cell-to-cell communication by enabling the measurement of the concentration and localization of high mannose-type N-glycans (HMs), which have different roles in a cell, such as the transportation of proteins from the Golgi apparatus to the various parts of the cells [45]. According to the intensity of HMs inside the microbeads, cell-to-cell and cell-to-microbead communication are elevated during 12 days of incubation (Figure 3B,C). However, there are no significant changes in the intensity of HMs inside the microbeads upon treatment with BET at 0.1, 0.5, and 1 µM on day 12. A statistically slight effect on HMs inside the microbeads was observed for 0.01 and 5 µM BET (Figure 3A,D).

### 3.5. Influence of BET on the Expression of Osteogenic Markers

**Figure 4:**
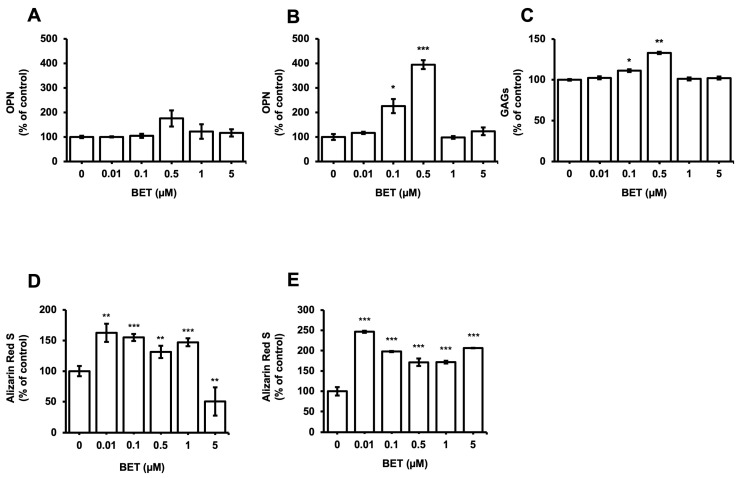
Expression of osteogenic markers in hFOB 1.19 cells after treatment with BET (0 to 5 µM). OPN levels in culture media of (**A**) hFOB 1.19 cells and (**B**) hFOB 1.19 cells encapsulated in AlGel microbeads incubated with different concentrations of BET (0 to 5 µM) on day 12. (**C**) GAG levels in culture media of hFOB 1.19 cells encapsulated in AlGel microbeads incubated with BET (0 to 5 µM) on day 12. (**D**) Calcium levels deposited in extracellular matrix of hFOB 1.19 cells in 2D culture and (**E**) hFOB 1.19 cells encapsulated in AlGel microbeads incubated with BET (0 to 5 µM) on day 12. *: *p* < 0.05, **: *p* < 0.01, and ***: *p* < 0.001.

The expression of osteogenic markers of encapsulated and non-encapsulated hFOB 1.19 cells was evaluated by measuring the OPN expression and glycosaminoglycans (GAGs). The production of OPN by osteoblast cells is a known marker of the differentiation process at the terminal stage. The expression of the OPN protein in encapsulated cells increased to 226% at 0.1 µM BET and 395% at 0.5 µM BET on day 12 (Figure 4B). There was no statistically significant increment detected in the expression of OPN in non-encapsulated cells. Similarly, the secretion of GAGs increased to 111% and 133% in encapsulated cells upon treatment with 0.1 and 0.5 µM BET (Figure 4C).

**Figure 5:**
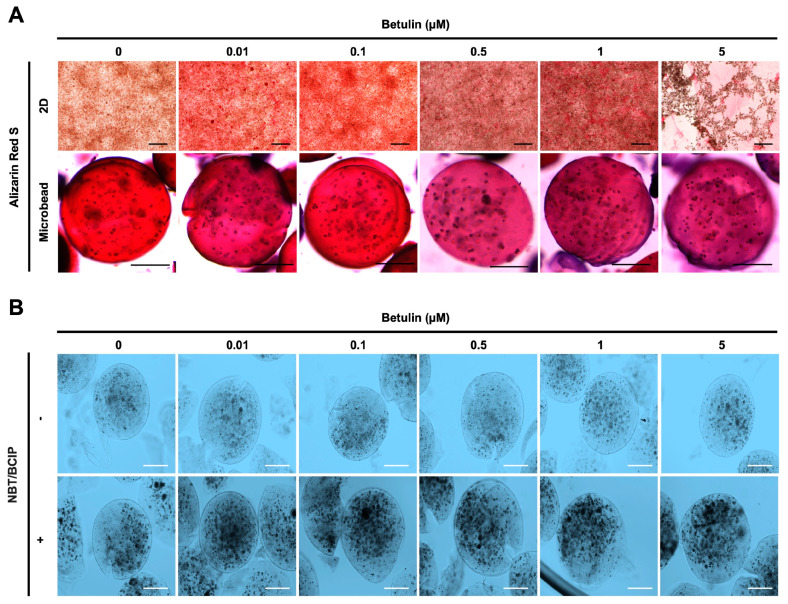
Effect of BET on mineralization and ALP activity of hFOB 1.19 cells. (**A**) Images showing calcium deposition of non-encapsulated (scale bar: 200 µm) and encapsulated (scale bar: 100 µm) hFOB 1.19 cells upon treatment with BET after 12 days of incubation using alizarin red S staining. (**B**) ALP enzyme activity of hFOB 1.19 cells in AlGel microbeads (scale bar: 100 µm) after 12 days of incubation using NBT/BCIP solution.

Moreover, the osteogenic differentiation of hFOB 1.19 cells potentially created the noodle formation, which was detected by alizarin red S staining at day 12 of incubation. Microscopy images of hFOB 1.19 cells clearly show an increase in noodle formations upon treatment with BET on day 12 (Figure 5A). The mineralization level of hFOB 1.19 cells was quantitively analyzed to assess calcium content after encapsulation and treatment with BET (Figure 4D,E). Alizarin red S staining indicated that BET treatment induced the osteogenic differentiation of hFOB 1.19 cells up to 163% at 0.01 µM BET. For encapsulated hFOB 1.19 cells, the BET treatment induced a much higher osteogenic differentiation, up to 246% at 0.01 µM BET (Figure 4D,E).

The osteogenic differentiation in encapsulated hFOB 1.19 cells was further assessed by measuring the ALP activity. Dark spots of ALP-positive microbeads indicate that enhanced ALP activity and early bone formation were successfully developed upon treatment with BET (Figure 5B).

## 4. Discussion

Alginate-based microbeads are commonly used in therapeutic delivery research and cell culture studies [46]. Previous studies have shown that such microbeads provide an excellent cellular ECM and 3D microenvironment to develop modular tissue using building blocks [6]. The incorporation of alginate and gelatin biopolymers enhances the performance and quality of the micro-scaffolds and simulates signaling cascades related to ossification, osteogenic differentiation, and the proliferation of cells [6]. The addition of gelatin to alginate microbeads enhances their potential for the proliferation and differentiation of osteoblast cells [6]. In addition, some triterpenoids, such as BET derivatives, improve the osteogenic differentiation of some murine cells in osteogenic conditions in vitro [37]. Moreover, porcine chondrocytes inside BET-treated scaffolds induce the expression of anabolic and catabolic genes and differentiation factors [47]. Although the osteogenic activity of BET on murine osteoblast models is confirmed in vitro, there are a limited number of studies examining the effect of BET and 3D microenvironments on osteoblast cell models for in vitro research. In this study, the osteogenic differentiation of hFOB 1.19 cell lines encapsulated in alginate–gelatin biopolymeric microbeads was examined after treatment with BET.

The gel strength of the calcium-crosslinked alginate decreased over the in vitro incubation time. The degradation of the microbeads occurs via the anion exchange mechanism and depends on the size, shape, porosity, and stability of the crosslinked alginate. Decreasing the crosslinking density over time results in disintegration of the alginate, weak hydrogel formation, and an increased pore size [48]. Increasing the pore size of the microbeads accelerates the inward diffusion of ions from outside of the microbeads, which leads to swelling [49]. However, the presence or absence of the cells corresponds to a slightly different microbead degradation over time. The rate of microbead degradation in the presence of cells gradually increases as the microbead concentration decreases, but changes over the 12-day period in the microbead diameter were not statistically significant. Microbead degradation in the presence of the osteoblast cells might be associated with native enzymatic activity or calcium uptake by osteoblast-like cells from the environment [50].

The anti-proliferative activity of betulinic acid was investigated in the encapsulated and non-encapsulated normal osteoblast cell line hFOB 1.19 model. The effect of BET on cell proliferation, osteoblast differentiation, and osteoclastogenesis has been described in in vitro research studies. There are various reports showing an alteration in the anti-proliferative activity of BET depending on the cell type. For example, while a 1 to 20 µM BET treatment on the murine pre-osteoblast cell line MC3T3-E1 does not significantly decrease cell viability [40], the proliferation of the human osteoblast cell (hFOB 1.19) and neoplastic osteoblast-like cell (Saos-2) were inhibited by BET at concentrations of 1 and 5 µM [41]. In addition, 10 and 20 µM BET treatments on the fifth day of culture promoted the proliferation of human periodontal stem cells (hPDLSCs) [51]. The observed cytotoxic effect of BET on hFOB 1.19 matches previous reports; however, no cytotoxic effect was observed on encapsulated hFOB 1.19 cells. Micro-encapsulation of hFOB 1.19 cells limits BET’s effect on the metabolic activity of the cells and provides the long-term functionality and survival of the cultured cells [43,52]. Thus, these results suggest that the encapsulation strategy is a powerful way to maintain the metabolic activity of cultured cells upon treatment with 5 µM BET.

The efficiency of injectable cell-based therapies on injuries depends largely on the viability of the transplanted cells post-injection. During the direct injection of stem cells, the syringe needle flow force disrupts the cell membrane and decreases the viability of transplanted cells at levels ranging from 1% to 32% [53]. The cell viability rate post-injection might be improved by the encapsulation of cells using hydrogels, which protect cells from the damaging effect of extensional forces by direct injection. In studies using alginate hydrogel as a scaffold for cell-based therapy, the composition of the alginate hydrogel not only modifies the chemical and physical properties of the hydrogel but also has an effect on cellular response and the efficiency of injectable cell-based therapies [6]. Many studies on the relationship between cellular response and hydrogels indicate that cell viability is enhanced because of the RGD-rich proteins from gelatin [18,28,54,55]. However, in vitro studies showed that the weakened structure of the polymeric materials and proliferation of cells inside the hydrogels limit the cell–material contact area and reduce the viability of encapsulated cells during cultivation [49,53]. Our results agree with the latter; the weakened structure decreases the cell viability rate inside AlGel microbeads from day 1 to day 12.

The formation of cells with a spherical or elongated form in the microbeads provides information about the interaction between the cells and materials. According to a previous report on cellular response to materials, the gelatin content inside an alginate mixture provides better cell adhesion properties because of the RGD (Arg–Gly–Asp) residue [19]. Cell-to-cell and cell-to-material communication maybe evaluated by the measurement of the level and localization of high mannose-type N-glycans (HMs), which have different roles in a cell, such as proteins transport from the Golgi apparatus to various parts of the cells. Additionally, cellular shape and cell-to-cell communication are regulated by the level of N-glycans inside the cell; for example, the accumulation of N-glycans leads to spheric forms [56]. Our findings on HMs support previous studies that show an increase of cell-to-cell and cell-to-material communication day by day inside AlGel microbeads [57].

Three stages are involved in the maturation of mesenchymal stem cells into osteoblast cells, followed by cell proliferation (condensation), extracellular matrix secretion, terminal differentiation with matrix calcification, and new bone formation. The fracture-healing process consists of the maturation of osteoblast cells, which proceeds from a series of cascade reactions and the expression of genes and markers. The level of genes’ expression indicates the progression stage of maturation in bone formation and bone defect healing. Activation of the signaling cascades by various agents, such as betulinic acid, induces the differentiation of mesenchymal stem cells into mature osteoblast cells [40,41,51,58]. BET promotes the upregulation of runt-related transcription factor 2 (RUNX2), which is responsible for the expression of osteoblast marker genes, such as ALP, osteocalcin (OCN), and osteopontin (OPN). Additionally, BET has a role in the regulation of glucose and lipid metabolism, and a beneficial effect on obesity via the activation of UCP-1 [59]. One study confirmed an opposing relationship between adipogenesis and osteogenesis via the identification of the mechanism of deacetylation of RUNX2 by Sirt-1, which is known as a major regulator of longevity and metabolic disorder. Resveratrol, for example, activates Sirt-1 and blocks nicotinamide (NAM), which inhibits RUNX2 and stimulates PPARγ for adipogenesis [60]. Similarly, BA induces the differentiation of osteoblast murine cells (MC3T3-E1) instead of the adipogenesis of adipocytes (3T3-L1) [61]. Consistently, betulin acid treatment at 0.5 µM on hFOB 1.19 cells significantly enhanced the expression of OPN, which is a late-stage differentiation marker. Moreover, the increased degree of calcification and calcified nodule formation of osteoblast cells after BET treatment support previous findings on mineralization [40,41,51,61].

Alginate microbeads stimulate the expression of osteogenic gene markers from adipose-derived stem cells, such as RUNX2 [49]. Alginate microbeads provide an appropriate environment to the bone cells for osteogenesis, the maturation of mesenchymal stem cells into osteoblast cells, and the stimulation of collagenous and non-collagenous protein expression in the ECM of the bone [62]. The chemical composition of alginate microbeads alters the functionality of cells and the efficiency of cellular therapy after a transplant process. For example, a bolus of calcium ions on the surface of alginate microbeads interacts with surrounding phosphate ions and results in calcification minerals, which are similar to hydroxyapatite (HA) [17]. Also, calcified microbeads have been obtained in vitro via the incubation of microbeads in DMEM, which allows the accumulation of PO_4_^−3^ and Ca^2+^ ions and the formation of a bone-like HA coating on the microbeads’ surface [63]. HA coating materials in vivo absorb various adhesive proteins, such as fibronectin from the serum solution [64]. Absorption of proteins by HA-coated materials makes polymeric surfaces more suitable for the migration and adhesion of osteoblast cells [65]. Similarly, HA inside alginate microbeads provides better gelatin binding to the microbeads and a more efficient environment for osteogenic cell proliferation and differentiation [6]. Moreover, HA content inside the microbead upregulates osteoblast marker genes such as osteopontin (OPN), osteocalcin (OCN), and RUNX2 [54]. The previous findings agree with our results: cells encapsulated in alginate–gelatin microbeads show a significant increase in osteogenic markers ALP and osteopontin (OPN) and in calcification, compared to a 2D cell culture.

Tissue-engineering studies lead to efficient cell therapeutics where the functional ability of cell transplants is maintained for early regeneration of the defect side. Cells require a supplement for the differentiation and maturation of a cell transplant to indicate the desired effect on the defect side [25]. Previous research on osteoblast cell-loaded alginate–gelatin microbeads and BET is promising for studies on the treatment of bone defects and osteoporosis. This study also agrees with previous reports and confirms that the rational and optimized treatment of osteoblast cell-loaded microbeads with BET may provide a more effective osteogenic in vitro proliferation and differentiation.

## 5. Conclusions

In this study, we investigated the osteogenic effect of BET on human fetal osteoblast cells (hFOB 1.19) in a 3D environment, where cells were encapsulated in alginate–gelatin polymeric microbeads. Our work reveals that the combination of osteoblasts with polymeric micro-encapsulation, and their treatment with supplements, improves the expression of osteogenic markers in in vitro conditions. The results also indicate that the 3D environment microbeads provide eliminates the negative effect of BET on the viability of osteoblast cells from 63% to 110% at a BET concentration of 5 µM. Furthermore, differentiation studies confirm that the microbeads promote a 2-fold increase in OPN secretion and a 1.3-fold increase in calcium deposition, compared to the 2D cell culture environment, at BET concentrations of 0.5 µM. Overall, this study demonstrates that stable and biocompatible polymeric microbeads provide a beneficial 3D environment which can contribute to accelerating the osteogenic effect of BET on the mineralization and differentiation of osteoblast cells.

## Data Availability

The data used to support the findings of this study will be available from the corresponding author upon request.
